# Heparin-Induced Renal Tubular Acidosis Masquerading as Hyperkalemia in a SARS-CoV-2 (COVID-19) Patient: A Case Report

**DOI:** 10.7759/cureus.20312

**Published:** 2021-12-09

**Authors:** Vidya Baleguli, Riaz Mahmood, Martin Herrera, Erine Raybon-Rojas

**Affiliations:** 1 Internal Medicine, Northeast Georgia Medical Center Gainesville, Gainesville, USA; 2 Critical Care Medicine, Northeast Georgia Medical Center Gainesville, Gainesville, USA

**Keywords:** covid-19, potassium, creatinine, renal tubular acidosis type 4, rta, hyperkalemia, heparin

## Abstract

Type 4 renal tubular acidosis (RTA) is a type of metabolic acidosis characterized by hyperchloremia and hyperkalemia resulting from the reduction in and/or resistance to aldosterone. RTA can be caused by multiple different medications including angiotensin-converting enzyme (ACE) inhibitor/angiotensin receptor blocker (ARB), potassium-sparing diuretics, and heparin. In this case, we discuss renal tubular acidosis caused by heparin use for the prevention of thromboembolic disease in COVID-19 infections.

## Introduction

The pulmonary and renal systems are the primary regulators of acid-base balance within the body. Through alveolar ventilation, the lungs can remove carbon dioxide from the body. Bicarbonate is filtered in the proximal and distal tubules along with the elimination of hydrogen ions [1,2].

Renal tubular acidosis (RTA) is defined as inadequate excretion of acids via the kidneys to maintain the acid-base balance. Metabolic acidosis develops as a result of the inadequate elimination of hydrogen ions or the impaired retrieval of filtered bicarbonate ions. The hallmark of all RTA is normal anion gap metabolic acidosis with hyperchloremia. In the case of type 4 RTA, also known as hyperkalemic RTA, there is either deficiency or resistance to aldosterone, leading to the impaired regulation of sodium, potassium, and hydrogen ions. This leads to hyperchloremia, hyperkalemia, and acidemia. Given this vital balance, we highlight an unsuspecting etiology of type 4 RTA as noted by heparin use for venous thromboembolism (VTE) prophylaxis in a SARS-CoV-2 patient [1,2]. Our case of heparin-induced RTA causing hyperkalemia in a SARS-CoV-2 patient has been presented at the CHEST Annual Meeting 2021.

## Case presentation

**Figure 1:**
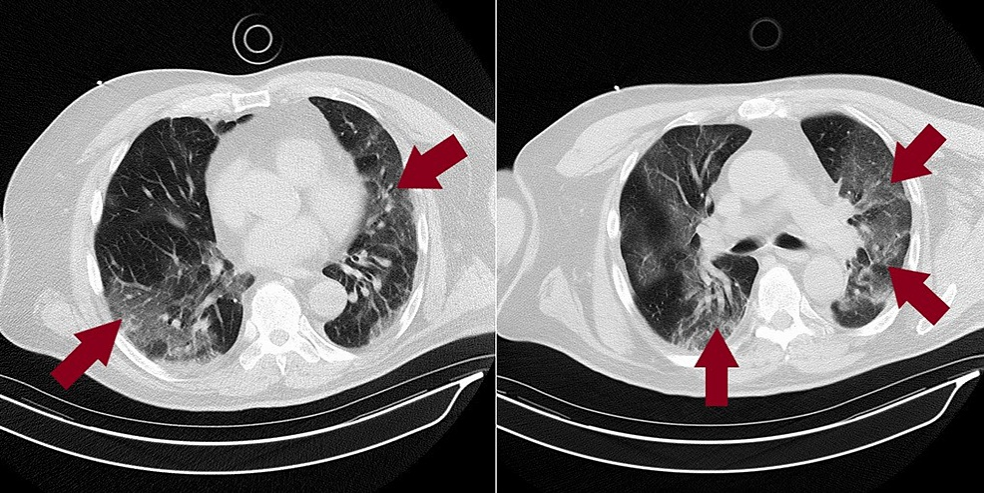
CT chest without contrast Bilateral diffuse nonzonal predominant airspace opacifications in the lung parenchyma consistent with manifestations of atypical pneumonia (maroon arrows) CT – computed tomography

A 72-year-old male with a past medical history significant for chronic kidney disease (CKD) and leukemia in remission presented with a six-day history of flu-like symptoms including intermittent diarrhea, fevers, decreased appetite, shortness of breath, and generalized malaise. The patient had exposure to several known sick contacts with SARS-CoV-2. At presentation, his vitals were notable for fever with a temperature of 102.3°F and respiratory rate (RR) of 21 breaths/minute. His oxygen saturation was 89% on room air initially but dropped into the low 80's subsequently. Laboratory work revealed blood urea nitrogen (BUN) of 24 mg/dL (normal range: 7-20 mg/dL), creatinine of 2.24 mg/dL with patient's baseline at 1.8-2 mg/dL (normal range: 0.74-1.35 mg/dL), estimated glomerular filtration rate (eGFR) 30.5 mL/minute/1.73 m^2^ (normal range: >60.0 mL/minute/1.73 m^2^), and white blood cell (WBC) of 12.3 K/uL (normal range: 4.5-11 K/uL), with absolute neutrophils of 11.33 × 10^3^/uL and absolute lymphocytes of 0.36 × 10^3^/uL. Arterial blood gas (ABG) showed pH of 7.463, pCO_2_ of 27.4, pO_2_ of 58 on 4 L/minute oxygen via nasal cannula, and lactate of 2.5 mmol/L. The patient continued to have oxygen desaturation requiring the use of a Venturi mask with subsequent improvement to 95%. Computed tomography (CT) of the chest without contrast showed bilateral diffuse nonzonal predominant airspace opacifications in the lung parenchyma, consistent with atypical pneumonia manifestations (Figure 1).

**Figure 2:**
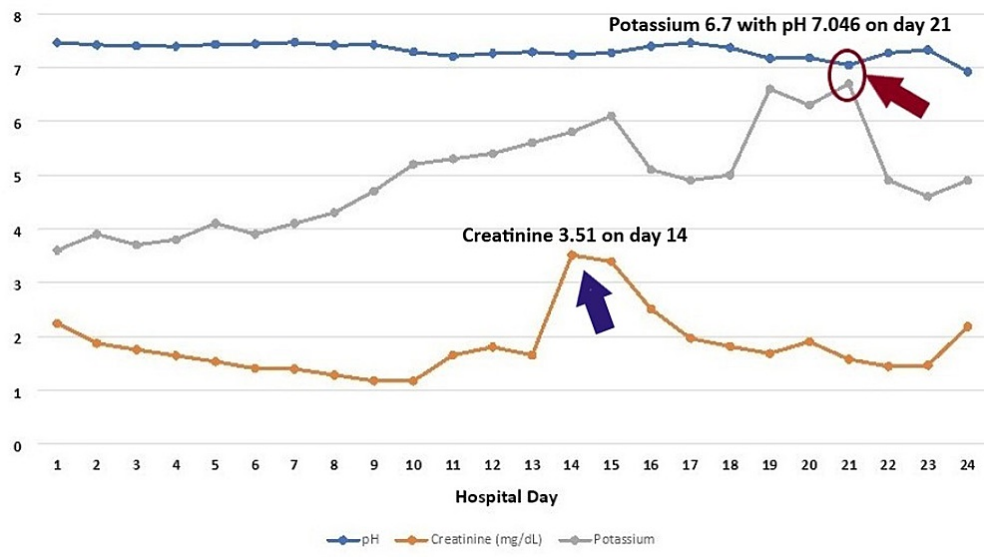
Graph showing the trend of creatinine, potassium, and pH Creatinine – mg/dL Potassium – mmol/L

The patient was admitted to the intensive care unit (ICU) for acute hypoxic respiratory failure and started on Decadron, remdesivir, azithromycin, ceftriaxone, enoxaparin, zinc, and vitamin C, and self-proning for at least 8-12 hours was encouraged. On hospital day 2, consent was obtained for convalescent plasma and transfusion. He continued to have worsening respiratory status due to which he was transitioned from high-flow to bilevel positive airway pressure (BiPAP), and Decadron was switched to Solu-Medrol 40 mg IV every 12 hours. The patient subsequently required emergent intubation on day 10. Creatinine up trended and peaked at 3.51 mg/dL on hospital day 14 (Figure 2). Urine analysis at that time showed hyaline cast, urine sodium 99 mmol/L, and urine creatinine 28.30 mg/dL.

The patient developed a new onset of atrial flutter, the rate was controlled, and he was continued on enoxaparin 80 mg BID. The patient was noted to develop hyperkalemia. Nephrology was consulted; the hyperkalemia was initially presumed to be secondary to acute kidney injury (AKI) superimposed on chronic kidney disease (CKD) stage 3b, which was likely from ischemic acute tubular necrosis (ATN) from sepsis compounded by obstructive uropathy from a nonfunctioning Foley catheter. His platelet counts declined by >50% more than 10 days after hospitalization as he was started on enoxaparin on day 1. His 4Ts score for heparin-induced thrombocytopenia (HIT) was 3-4. Due to concern for HIT, argatroban was started but subsequently stopped due to bleeding. As fondaparinux is contraindicated in renal failure, he was started on heparin infusion on day 15 when the HIT ab (-) result was obtained.

His serum potassium continued to trend up and peaked at 6.7 mmol/L on day 21 during which the pH was noted to be 7.046 (Figure 2). He was treated on calcium gluconate, insulin, Dextrose 50, and patiromer 8.4 g with no significant improvement in potassium levels after multiple days of treatment even though the creatinine levels normalized. The discrepancy between the creatinine peak, which was on day 14, and the potassium peak, which was on day 21, further reassures us that the hyperkalemia is not secondary to AKI on CKD stage 3b. The ongoing acidosis with a pH of 7.046, normal anion gap of 5 mmol/L (normal range: 4.3-12.3 mmol/L), and chloride of 113 mol/L (normal range: 100-110 mmol/L) at the time of potassium peak is the hallmark of all RTA, which is the normal anion gap metabolic acidosis with hyperchloremia. Due to concerns that the use of heparin may be causing hyperkalemia by suppressing aldosterone, heparin was discontinued. Subsequently, serum potassium levels improved to the normal range (Figure 2).

He was noted to have deteriorating acidosis with ABG showing pH of 6.923, pCO_2_ of >102 mmHg, and pO_2_ of 83 mmHg with the inability to maintain adequate saturations despite the manipulation of the ventilator. Attempts at correction of acidosis were unsuccessful with intravenous fluids containing bicarbonate, and dialysis was being considered if the family planned to continue with aggressive treatment. He was noted to have worsening leukocytosis and was started on cefepime and Zosyn for *Enterococcus faecalis* and *Staphylococcus epidermidis* bacteremia per the blood cultures. Subsequently, a decision was reached by family members to withdraw care. The patient was terminally extubated on day 24.

## Discussion

The renal system is under the influence of aldosterone to properly regulate sodium, potassium, and chloride. Aldosterone acts on principal cells to reabsorb sodium via epithelial sodium channels (ENaCs) and secrete potassium via potassium channels. This regulation is further assisted by the sodium-potassium ATPase on the basolateral surface of principal cells. In type 4 RTA, either from low levels of aldosterone or from the kidneys not responding to aldosterone, impaired sodium reabsorption and potassium secretion occur. The impaired sodium reabsorption leads to disturbance in the electronegativity across the tubular lumen. This impairs distal acidification as the driving force for hydrogen ion secretion is lost [2].

In a patient, like the one encountered in our case, with persistent hyperkalemia on heparin, in whom there is no apparent cause such as the use of potassium supplements or a potassium-sparing diuretic or renal failure, heparin-induced type 4 RTA must be considered [3-5]. Type 4 RTA may also stem from diseases that modify the kidney structure and function such as diabetic nephropathy, HIV/AIDS, Addison's disease, sickle cell disease, urinary tract obstruction, lupus, amyloidosis, removal or destruction of both adrenal glands, and kidney transplant rejection. The medications that may cause type 4 RTA include diuretics such as spironolactone or eplerenone, angiotensin-converting enzyme (ACE) inhibitors/angiotensin receptor blockers (ARBs), trimethoprim, pentamidine, heparin, and nonsteroidal anti-inflammatory drugs (NSAIDs) [2].

In COVID-19 patients, there exists a higher propensity for higher rates of thromboembolic disease and often prevention with heparin can serve as the best treatment. Heparin has a direct toxic impact on the adrenal zona glomerulosa cells, which may be facilitated by a drop in the number and affinity of adrenal angiotensin II receptors [4,6]. Even at lower doses, heparin given twice daily at 5000 units can cause a significant reduction in plasma aldosterone concentrations [7]. This reduction in aldosterone can then lead to severe hyperkalemia especially in the setting of renal insufficiency, concomitant use of ACE inhibitor/ARB, or potassium-sparing diuretics [6].

Furthermore, even lower molecular weight heparin can have a similar effect on potassium regulation. For instance, Canova et al. reported eighty-one patients treated with low molecular weight heparin for prevention of venous thromboembolism (VTE) were noted to have higher potassium plasma concentrations. Approximately 5 to 10% increase in potassium can be noted and the highest increase, from 5.11 to 5.70 mmol/L of potassium, was noted in a patient with renal failure [8]. Similar findings have been reported in other publications [9].

## Conclusions

This case is unique because hyperkalemia was noted to be refractory to the typical standard treatment. It was subsequently noted that the reason for this resistance was hypoaldosteronism likely secondary to heparin use. Most patients with RTA if treated adequately in the preliminary stages do not develop permanent kidney failure and have better mortality and morbidity outcomes.
